# Resilience in middle-aged partners of patients diagnosed with incurable cancer: A thematic analysis

**DOI:** 10.1371/journal.pone.0221096

**Published:** 2019-08-14

**Authors:** Sophie Opsomer, Peter Pype, Emelien Lauwerier, Jan De Lepeleire

**Affiliations:** 1 Department of Public Health and Primary Care, KU Leuven, Leuven, Belgium; 2 Department of Public Health and Primary Care, UGent, Gent, Belgium; 3 End-of-life Care Research Group, VUB & UGent, Gent, Belgium; 4 Department of Experimental-Clinical and Health Psychology, UGent, Gent, Belgium; University of Technology Sydney, AUSTRALIA

## Abstract

**Background:**

Providing care for patients with advanced cancer is often the responsibility of the partner. Being confronted with an incurable cancer diagnosis can be highly disruptive for the patient’s partner and can be considered a potentially traumatic event. However, most caregivers seem to adapt well during the process of providing care. This finding is in line with the concept of resilience in literature: a dynamic process of adapting well, resulting from the interplay between intrinsic and extrinsic resources and risks. Resilience is age-related, with the elderly population being higher in resilience as compared to the younger generation. However, resilience has been understudied in middle-aged caregivers.

**Aim:**

To explore what intrinsic and extrinsic resources facilitate or hamper resilience in the middle-aged partner of a patient with incurable cancer.

**Methods:**

Nine middle-aged partners of patients who died at home of cancer were selected and interviewed in depth within the first year following the death of their partner. A thematic analysis utilizing an inductive approach was conducted.

**Findings:**

Resilience was challenged by the partner’s diagnosis of incurable cancer. All participants made use of a set of interacting, caregiver-specific and context-related resources, facilitating a resilient process and leading to positive feelings and even personal growth. The partners demonstrated individual competences: adaptive flexibility, positivism, a sense of self-initiative and adaptive dependency. Furthermore, they relied on their context: cancer-related professionals and relatives. Context and situation interact continuously. The resulting dynamics were based on the context-availability, meaningful relationships and the patient’s role.

**Conclusion:**

A resilient trajectory results from an interplay between individual and contextual resources. To build resilience in middle-aged partners of patients with incurable cancer, health care professionals should address all available resources. Moreover, they should be aware of being part of the caregiver’s context, a complex adaptive system that can be either resilience-supporting or -threatening.

## Introduction

Cancer incidence worldwide has risen by 28% during the past decade, and in 2016, 8.9 million cancer deaths were reported [1]. Additionally, the impact of being diagnosed with an advanced and incurable cancer expands far beyond the patient, affecting his/her close family and friends [2, 3]. Moreover, the ongoing shift from inpatient to outpatient care for patients with advanced cancer has been possible by the tireless commitment of the informal, unpaid caregivers, often the patient’s partner [3]. Being confronted with a partner’s terminal cancer diagnosis, can be considered a highly disruptive, and as such, potentially traumatic event (PTE) [4, 5].

Research on a PTE in general and on caregivers of patients diagnosed with advanced cancer in particular, has been dominated by a pathophysiological approach, focusing on a negative outcome [6]. These studies reveal that the threat of a partner’s pending death, the sheer number of new responsibilities and tasks to undertake, and the financial consequences of the disease, often lead to a considerable burden to the caregiver, possibly resulting in suboptimal health. Being the caregiver of a patient diagnosed with cancer often comes with an increased risk of major depressive disorders, anxiety, decreased ability to concentrate, decline in health-related quality of life, loss of sleep, loss of appetite and social isolation [7–14]. Large studies in caregivers of patients diagnosed with cancer, report depression-rates between 5,1–41,8%, while anxiety was diagnosed in 17,9–42,2% of cancer caregivers [8, 14–17]. The anxiety and depression rates were significantly higher in caregivers than in the general population [14, 16].

Longitudinal studies about anxiety and depression among caregivers are scarce. Tang et al (2013) evaluated depression in 193 caregivers of terminally ill cancer patients over time. They conclude that depressive symptoms increase from 45,8% to 54,9% in the last month before the patient’s death and with increasing symptom burden. Lee et al. assessed 132 caregivers of patients with head and neck cancer over a 6-month follow-up period. They state that both depression and anxiety rates decrease over the first 3-month follow-up (from 14.7% to 14.6%) and lower further in the next 3 months (12.9%) [18].

Nevertheless, despite the burden, the largest group of caregivers seem to adapt well to the new condition, a process that is called resilience [19–23]. Numerous studies explore anxiety and depression in caregivers. However, studies starting from a positive viewpoint, resilience for instance, are scattered. Consequently, when describing the characteristics of the resilient trajectories, we have to rely on studies about resilience in other groups or after other PTE’s (e.g. in bereaved caregivers) and we can only assume that caregivers of cancer patients will follow the same trajectories as described after other PTE’s or post-loss.

People who follow a resilient trajectory, express positive emotions, and report having an adequate health-related quality of life [4, 21–24]. Hence, resilience seems to buffer against mental health problems. More than half of the caregivers even report positive consequences of caregiving, for example, reciprocating favors, experiencing closer family relationships or feeling accomplished [25–27]. This is in line with the results of prospective studies and reviews about adaptation after different kinds of PTE’s [6, 24, 28–36], assuming that, after exposure to a PTE, a resilient trajectory is normative [6, 28, 34, 35] as most people seem to be able to adapt well, and after a short-term mild or moderate stress reaction, emotional pain, or sadness, regain a stable equilibrium and move on to the new challenges without symptoms of depression or post-traumatic stress disorder [24, 30, 34, 35, 37].

Galatzer-Levy and Bonanno (2012), for instance, examined prospective trajectories of response to bereavement from pre-loss to four years post-loss on a data-set obtained by 205 widowed persons. They observed that 66,3% of the participants followed a resilient trajectory, identified by absence of depression or very low depression scores from before the loss to four years after the loss. Fourteen and a half percent of the participants were chronically depressed without improvement in the depression scores while chronic grief, identified by high levels of depression during 18 months, followed by a steadily returning to pre-loss levels, was apparent in 9,1%. Participants with high pre-loss depression scores (10,1%) returned to pre-loss scores within the first six months post-loss and were referred to as depressed-improved. These results are similar to other prospective studies investigating trajectories after a PTE [28].

Bonanno et al (2015) reviewed the literature about sequential models of resilience. From studies about acute adversities such as terrorist attack, physical assault, an isolated medical emergency, loss of a spouse, heart attack, chronic pain onset and life-threatening medical events, such as receipt of a cancer diagnosis. They conclude that a PTE is most commonly followed by minimal-impact resilience meaning that resilient individuals apparently adapt well and endeavor to overcome the temporary disruptions caused by the PTE (e.g. symptoms of distress, pre-occupation or restless sleep), over a period no longer than one month [38]. These findings were recently confirmed by a systematic review of prospective and longitudinal studies that investigated the trajectories following a PTE (civilian trauma, rape, war, military deployment, heart attack, cancer diagnosis, loss and spousal bereavement, spinal cord injury or chronic pain onset). The prevalence of the resilience trajectory depended on the event type, was independent of the severity of the PTE and remained high even after multiple PTEs [34].

Over the years, the concept of ‘resilience’ has been approached by a variety of academic fields and disciplines, such as economics, engineering, psychology, sociology and nursing, and consequently, has been formulated in just as many ways [36, 39]. Although there is no consensus on an unequivocal definition of resilience, experts appear to largely agree on two aspects: the individual should 1) be exposed to adverse conditions, either chronically or by a single PTE; and 2) be able to adjust positively despite adversity [40, 41]. In this study, the definition of the American Psychological Association (APA) is adopted: *Resilience is the process of adapting well in the face of adversity*, *trauma*, *tragedy*, *threats or significant sources of stress. It means ‘bouncing back’ from difficult experiences*. [37].

This definition may conveniently be linked to the dynamic framework of Kumpfer (1999) and Bonanno (2015). Both accentuate the interaction between internal and external resources, the influence of the stressor, the coping processes that are adaptable (these can be learned through repetitive exposure to challenges) and the successful outcome, suggesting resilience [38, 42].

Resilience has been extensively explored in children living in chronic adverse circumstances (i.e., children raised in poverty or confronted with family violence) [41], as well as in adults following a PTE, like bereavement, terrorist attack or serious illness [5, 30, 43–45].

Resilience has scarcely been addressed in caregivers of patients with advanced cancer[46–50]. Moreover, research results of studies on anything other than cancer caregiving situations (e.g., dementia caregiving or chronic illness), cannot simply be transferred onto caregivers of patients with cancer because of the very specific situation and the interaction between context and resilience [42, 51].

It is commonly-known that resilience is age-related, with people 65 years or older, showing levels of resilience three times higher than that of younger people [52]. Moreover, middle-aged caregivers more often develop depression and anxiety. Presumably, this is due to their specific context, which is often characterized by an unstable financial situation [2, 51, 53]. However, resilience has never been studied in this group of caregivers, as far as we know.

To further explore the concept of resilience in an advanced cancer caregiving context with emphasis on the experiences and views of the caregivers themselves, an inductive, qualitative inquiry with a thematic analysis study design was launched. Qualitative data, deriving from caregivers’ real-life experiences can provide a valuable source of information, insights and new ideas about supporting and challenging resilience in caregivers.

Insight into the intrinsic and extrinsic resilience-supporting or -threatening features can be beneficial to nurses, psychologists and other health care professionals. More specifically, it can help them to recognize caregivers who will most likely experience a resilient trajectory and those who are at risk of experiencing a negative outcome such as depression, anxiety or even PTSD.

Resulting from the gaps in knowledge described above, the aim of the current study is: to explore what intrinsic and extrinsic resources facilitate or hamper resilience in the middle-aged partner of a patient with incurable cancer.

## Methods

### Research team and reflexivity

The first author, a family physician experienced in palliative care and in qualitative research, initiated the study and conducted the interviews as part of her PhD project. She had no professional relationship with the candidates and did not meet the candidates until the day of the interview. Two authors are professors in primary healthcare, and one is a doctor in clinical psychology. All members of the research team are experienced researchers in the field of palliative care and communication in healthcare. Except for the interviewer, none of the authors were in contact with the participants.

### Study design

#### Theoretical framework

To explore the concept of ‘resilience in a cancer caregiving experience’, a thematic analysis with an inductive approach was chosen. This method is suitable for exploratory work in an understudied area and provides a rich, detailed and nuanced account of the data [54, 55].

#### Participant selection

Principal caregivers of recently deceased middle-aged cancer patients were recruited from their client list by two Flemish Palliative Home Care Teams. An invitation to participate, including written information about the study, was sent to 85 caregivers who met the inclusion criteria: having been the principal caregiver of a patient who died because of cancer less than one year ago at an age between 40 and 59 years old; and a resident of Flanders and fluent in Dutch. Eighty-five caregivers met the inclusion criteria. Twenty-two caregivers contacted the researcher to let her know that they were pleased to participate. No letters with a reason of non-participation were returned. To maintain homogeneity in the group, only middle-aged (40–59 years) co-habiting life partners of the patient were selected, resulting in fourteen eligible candidates for participation unless prior data saturation was achieved.

#### Data collection

A semi-structured interview guide was developed, pilot tested and adapted. The data from the pilot interview were not included in the analysis. The emphasis of the interview guide was on encouraging the participants to provide a narrative account of the caregiving experience. Predefined questions and probes were employed to encourage caregivers when necessary. After the analysis of the first three interviews, the interview guide was adapted to the emerging themes, and consequently, was refined after each interview. The primary topics are represented in Table 1.

**Table 1:** Interview guide, adapted to the emerging themes after the third interview.

• Could you tell me something about the time when you were your husband’s or wife’s caregiver? What hit you the most? • What made you decide to take up the role of caregiver? • What expectations did you have? Did it turn out the way you expected? • Did you talk about being a caregiver with friends, colleagues or professionals, before taking up the caregiving role? What did they advise you? • Did you ever consider bringing your husband/wife to an inpatient palliative unit? What made you decide (not) to do so? • What situations where the most difficult? How did you manage them? • Could you tell me more about the difficulties you experienced as a caregiver? • What helped you to cope with this? • In what situations did you tend to lose control? What did you do in such situations? • What positive memories do you have? • Could you tell me more about the positive aspects experienced while taking care of your husband or wife? • If someone in a similar situation would ask for your advice about taking up the role of caregiver or not, what would you advise? • Looking back on the caregiving period, do you consider it positive or negative? What aspects made it a positive or a negative experience?

To ensure that the whole spectrum of experiences was covered by the themes discovered, the participants were encouraged to share their full stories. They were asked about different aspects of positive psychological concepts and their facilitators, as well as about threats or difficulties. Nevertheless, interviews 7, 8 and 9 did not reveal new information. Hence, no new codes could be added to the code tree, and data saturation was presumed after the sixth interview. The data saturation table is presented as Table 2.

**Table 2:** Data saturation table.

Interviews		I1	I2	I3	I4	I5	I6	I7	I8	I9
Codes	Staying active and socially engaged		X							
	Expressing positive emotions		X							
	Searching for information or practical solutions	X								
	Controlling the situation	X								
	Coming into action	X								
	Keeping the patient out of the hospital				X					
	Fending off negativity				X					
	Accepting the situation being unique				X					
	Actively seeking help and support		X							
	Accepting help and advice		X							
	Acquiring new skills			X						
	Accepting a role one did not choose			X						
	Adapting lifestyle		X							
	Taking some time for oneself		X							
	Couple activities	X								
	Giving positive meaning to the crisis		X							
	Positive feelings					X				
	Finding benefits	X								
	Personal growth						X			
	Fulfilling the patient’s wishes		X							
	Sustaining a sense of hope		X							
	Maintaining normality				X					
	Availability of HCPs		X							
	Connectedness with friends and family		X							
	More intense relationships		X							
	The strength of the family		X							
	Quality of the relationship		X							
	A happy patient		X							
Number of new codes per interview		5	15	2	4	1	1	0	0	0
Cumulative number of codes		5	20	22	26	27	28	28	28	28

I = interview; HCP = health care professional

X indicates in what interview the code was first expressed

The interviews were conducted between June 9 and October 14, 2017. They took between 42 minutes and 2 hours and 20 minutes, with an average of 75 minutes each. All interviews were audio recorded and transcribed verbatim, including non-verbal signals (e.g., caregiver looking away from his wife’s photo; crying, difficulty in speaking, lowering of voice) by the interviewing researcher.

#### Setting

All caregivers preferred to be interviewed at home. Only the interviewer and the caregiver were present.

#### Data analysis

Nine interviews were thematically analyzed. All members of the research team took part equally in the analysis process. A thematic analysis searches for patterns within data by organizing and describing the data set and by interpreting various aspects of the research topic [55]. After analysis of the fourth interview, the first and second authors discussed the preliminary findings to fine-tune the level of interpretation.

Before the start and throughout the analysis process, some methodological issues were discussed within the research team and the following decisions were taken:

- Only repeated patterns, detected by an iterative in- and cross-case search, could be considered a theme.
- There must be consensus within each theme, ultimately providing important information regading the research question. If a theme was not relevant to the research question, it was deleted.
- An inductive approach was preferred: being a middle-aged partner of a patient with incurable cancer is rather an unexplored situation, making it plausible that existing frameworks could not directly be applied to the study data [56].
- In this thematic analysis, a comprehensive stance was taken, focusing on both semantic themes directly derived from the data and latent themes requiring more interpretative work.

The actual thematic analysis of the transcripts was a team process consisting of six steps as suggested by Braun and Clarke [55, 57].

Step 1: All authors have read and re-read the interviews, meanwhile searching for patterns and meanings. Immediately after each interview, the first author wrote narrative accounts of the participant’s story, providing an initial list of ideas and points of interest. All narratives were discussed among the research team.
Step 2: Initial codes were generated iteratively by transferring interesting features reported by the participants to a more conceptual level.
Step 3: Themes were developed from the initial codes by clustering relevant data. Eventually, themes were retained or rejected by consensus. The themes and codes were then introduced in NVivo 12 by the first author. Quotes were linked to the codes.
Step 4: A hierarchical map, including concepts and themes, was developed by the research team. Each theme was tested for accuracy, and the map was reviewed for the representation of the ideas and meanings of the entire data set.
Step 5: The mapped themes underwent further defining and refining.
Step 6: The themes and the underlying story were described and completed with illustrating quotes.

### Ethics

Interviewing the recently bereaved can be either an emotional burden or a therapeutic talk [58, 59]. The interviewer—a medical doctor experienced in palliative care and in communication in healthcare—immediately addressed strong emotions as they arose during the interview. If necessary, the recording was paused, and the participant was offered to talk about their feelings and to end the interview. The interviewer provided her e-mail address and could be contacted afterward.

Ethical approval was provided by the Ethical Commission of University Hospitals Leuven on May 5, 2017. The study number is S60383.

Written informed consent was obtained from all participants.

### Validity and reliability

To increase credibility and reliability, each interview was followed by a debriefing of the participant and a brief peer debriefing of the interviewer with the study supervisor [57]. Field notes were made during and immediately after each interview to assure reflexivity [57]. The thematic analysis was conducted in a structured and traceable way. The appropriateness of the codes was verified by in-case and across-case analysis, a rigorous collaborative team process increasing confirmability [57].

## Findings

Participants’ demographic characteristics are represented in Table 3.

**Table 3:** Patients’ and their co-habiting partners’ demographic characteristics.

	Patient				Partner					
	Age at death	gender	diagnosis	Duration of illness from diagnosis to death	Age	Gender	Marital Status	Education	Lenght of caregiving period	Number of children living at home
C1	50s	Male	Colon Cancer	>5 years	50s	Female	Married	Higher Education	>5 years	0
C2	50s	Male	Glioblastoma	<1 year	50s	Female	Married	Higher Education	<1 year	1
C3	50s	Male	Melanoma	1–2 years	50s	Female	Married	Higher Education	1–2 years	0
C4	50s	Female	Breast Cancer	>5 years	50s	Male	Married	Higher Education	<1 year	3
C5	50s	Male	Pancreatic Cancer	<1 year	50s	Female	Married	Higher Education	<1 year	0
C6	50s	Male	Colon Cancer	1–2 years	50s	Female	Married	Higher Education	1–2 years	2
C7	50s	Male	Glioblastoma	2–3 years	50s	Female	Married	Higher Education	2–3 years	0
C8	40s	Female	Melanoma	2–3 years	50s	Male	Living together	Higher Education	<1 year	1
C9	50s	Female	Colon Cancer	1–2 years	40s	Male	Married	Higher Education	1–2 years	3

C = caregiver/partner

All patients were cared for at home until death. Most were diagnosed with an incurable cancer without ever having been diagnosed with cancer before. Their partners immediately took up a caregiving role. Two patients were diagnosed with cancer at an earlier stage and did not need a caregiver until the cancer was incurable. Eight of them received professional homecare from no less than three professionals–a community nurse, a GP and a palliative nurse. The caregivers are referred to as C1-9.

All our participants’ stories started with a PTE, namely, the partner being diagnosed with an advanced cancer in an incurable stage.

Nevertheless, all partners adapted well and followed a resilient trajectory characterized by managing or mastering such a challenging situation. Managing included controlling or altering the situation mostly by a straightforward way of coping, characterized by situation-corrective actions. Mastering included accepting that the patient’s life was coming to an end and flexibly adjusting lifestyle to the situation.

**Fig 1:**
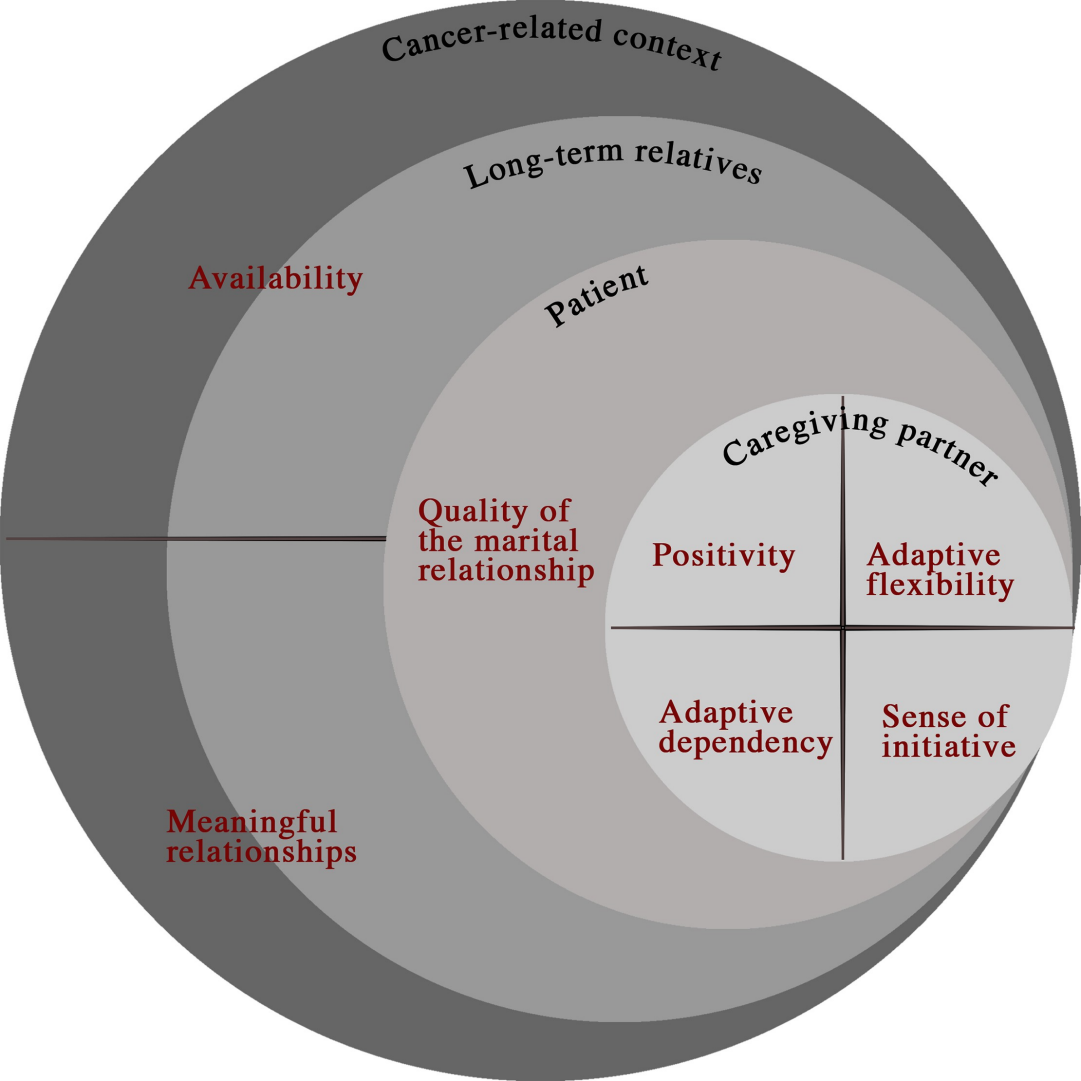
Intrinsic and extrinsic resilience facilitators in middle-aged partners of patients diagnosed with incurable cancer.

From our data, two categories of facilitators of a resilient process could be distinguished. They are presented graphically. (Fig 1)

- category 1: caregiver resources comprising four themes: sense of initiative, adaptive dependency, adaptive flexibility and positivism.
- category 2: context resources comprising three themes: availability, meaningful relationships and the patient’s role.

The hierarchical map, including concepts and themes, is presented in Table 4

**Table 4:** Hierarchical map including concepts and themes.

	Themes	Codes	Definition
**Caregiver resources**			The caregiver’s competences facilitating a resilience trajectory characterized by either managing or mastering the challenging situation.
	**Sense of self-initiative**		Caregivers exposing sense of self-initiative are striving towards having everything under control and being prepared for emerging situations. They are ceaselessly working towards reaching their goals.
		Searching for information or practical solutions	
		Controlling the situation	
		Coming into action	
		Keeping the patient out of the hospital	
		Fending off negativity	
		Accepting the situation being unique	
	**Adaptive dependency**		Caregivers exposing adaptive dependency are willing to seek help and support to resolve their practical problems or to handle their emotional disruptions, even when the situation mainly demands individual functioning.
		Accepting help and advice	
		Actively seeking help and support	
	**Adaptive flexibility**		Caregivers exposing adaptive flexibility are willing to adopt a new role and learn new skills. They adapt their lifestyles or look for distraction when they need to do so.
		Acquiring new skills	
		Accepting a role one did not choose for	
		Adapting life style	
		Taking some time for oneself	
		Couple activities	
	**Positivism/optimism**		The caregiver succeeds in giving positive meaning to the crisis. He or she sustains a sense of hope and trust.
		Fulfilling the patient’s wishes	
		Staying active and socially engaged	
		Expressing positive emotions	
		Giving positive meaning to the crisis	
		Positive feelings	
		Finding benefits	
		Sustaining a sense of hope	
		Maintaining normality	
**Context resources**			
	**Availability**		Knowing people are available day and night.
		Availability of HCPs	
		Availability of friends and family	
	**Meaningful relationships**		Being part of a system consisting of interacting with relatives.
		More intense relationships	
		Connectedness with friends and family	
		The strength of the family	
	**The patient’s role**		Reciprocity and mutuality in the performance of the caregiver and the patient.
		Quality of the partner relationship	
		A happy patient	

### Caregiver resources

Themes in this category refer to the facilitators related to the ability to adapt to the new situation and to facilitate the resilience process.

#### Sense of self-initiative

Caregivers who exposed a sense of self-initiative were working towards controlling current and potential situations. They ceaselessly worked in order to reach their goals (e.g., keeping their partner alive as long as possible).

For example, when C1 heard there were no curable therapies left, she got on the Internet and finally was able to have her husband accepted into a chemotherapy program in a neighboring country.

C1: *I knew it was available in a neighboring country*, *so I e-mailed the firm and called the local representative in advance to inquire which doctors had contributed to the study*, *and so on*. *Then*, *I contacted them*. *I received e-mail addresses*, *and we got accepted abroad*.

Some caregivers considered the hospital as the place where life ends. Pursuing their goal of keeping the partner alive, they made their best efforts to keep their partner out of the hospital.

C4: *And she was often hospitalized*. *You can’t do anything about it*, *right*? *It’s difficult to go there*. *It’s difficult to be there*. *I sat there for days*, *but you can’t do anything*. *Even you stop living*. *(*…*) Her sister came to relieve me*, *but you can’t do anything*. *Life stops there*. *That’s not the case at home*.

Caregivers with a sense of self-initiative accepted the situation being a challenge instead of a crisis and took action.

C2: *I immediately took action*. *Because we immediately knew that* … *well* … *that it wouldn’t go well anymore*. *They told our family one year*, *maybe one year and a half*. *Of course*, *I immediately took action*. *(…) I should say that I think*, *we are not people who just keep waiting for something to happen*.

However, when challenges were deemed to be an invincible obstacle to reaching the goal, the caregiver found a way to overcome the barriers by fending off negativity. C1 and C4, for example, experienced talking about partners who were dying, removing all hope for a long life together. Health care professionals or friends who wanted to talk about the end of life or who came to say goodbye found the door locked.

*C4: And then the palliative homecare team that regularly called and wanted to come by. But, for myself, I decreased those two. We haven’t actually built such a good relationship with the palliative team. Every time they came, they had bad news, and those people are focused on that, right*.

Self-initiative caregivers were aware of their “existential aloneness”, experiencing the cancer as an unshared story. Despite all of their family and friends’ good intentions, they would finally have to deal with the cancer themselves.

C9: *We were such independent people who had our lives in order*. *So*, *you’re not used to that*. *We weren’t used to asking for help*, *we never needed it*. *It wasn’t our lifestyle to ask for help*, *we did everything on our own*. *We only asked for help from each other*. *The two of us could handle everything*. *(…) That her illness was our little thing*. *Yes*. [laughs]. *Yes*. *We also did it with just the two of us*.

#### Adaptive dependency

Caregivers exposing adaptive dependency were willing to seek help and support to resolve their practical problems or to handle their emotional disruptions, even when the situation mainly demanded individual functioning.

Not knowing what was going to happen nor what they were expected to do when their partner died was a tremendous threat to most caregiver’s resilience.

C2, for example, tried to overcome the uncertainty and anxiety about what was going to happen when her husband died by looking for emotional support and asking her peers about their experiences.

C2: *I then called on someone we know well*, *a good friend*, *whose wife also died at home*, *after the whole process at home and*, *by chance*, *the same GP*. *I called him asking*, *should I do that*? *Am I able to do that*? *Because*, *like*, *you’re afraid of that too*, *right*. *How is this all gonna go*? *And the dying*, *how’s that gonna go and will I be able to handle that*? *There are so many questions going through your head*.

Although caregivers displaying adaptive dependency welcomed all offered help and advice, the advice given by her mother-in-law was skeptically accepted by C6 since it made her feel uncertain, even a failure.

C6: *And she brought food and stuff*, *and sometimes I had this feeling that I wasn’t performing well enough anymore*. *Although her intentions were good*, *it came across as… well*, *sometimes you almost felt guilty*, *as if she thought the illness was because of me… Because she told me*, *for example*, *how I had to prepare the fish and that it had to be frozen first* … *She began to tell me how to do it all*.

#### Adaptive flexibility

Most participants adapted flexibly to the new situation by adopting a new role, learning new skills, changing their lifestyle or looking for distraction when they needed.

C3, C4, C5 and C8 either took up a nursing role, learned how to administer enteral feeding or were trained in using a PICC-catheter. C2, C4, C7 and C9 took over tasks that used to be done by the patient, while C7 described herself as “the patient’s cab-driver”.

C3: *I went there in agony with him*. *But I did it (…) And there*, *in that foreign country*, *with those techniques*. *Because they didn’t know the technique of draining the fluid*, *they taught me how to do it in the hospital*, *so I could do it myself*. *And uhm*, *I did do it*, *but it was so difficult because I was in a different role there*. *In the end*, *I really was his nurse*. *(…) Then we drove back home*. *I had never driven that long myself*. *I didn’t even like to drive with his car because it was so big*. *Actually*, *I don’t like to drive at all*. *But I didn’t have a choice back then*. *So*, *I had to overcome several fears*, *but in such a situation*, *you just do it*.

Couples adapted their lifestyle to increase the patient’s comfort (e.g., by sleeping downstairs or by taking a cab instead of the subway). At the last minute, C8 changed the means of transport because his partner was not allowed to fly.

C8: *Yes*, *sometimes she couldn’t do this*, *she couldn’t fly for example (…) Normally*, *we would plan to leave on Saturday*, *and on Friday they told us that she was not allowed to fly*. *So*, *alright*, *fine*, *then we just go somewhere else with the car*.

Adaptive flexibility was also expressed in the way caregivers sought out an equilibrium between caretaking and searching for distractions by taking some leisure time for themselves or by going to work for a few hours each day.

C9: *Because my job became my distraction*. *And right when I had to start working in that period*, *shortly after the chemo started*, *it became very busy again*. *That became my biggest distraction*. *(…) And then you have a trip abroad once every two weeks*. *When I was over there for example*, *I walked*, *ate something with a beer*, *drank a bit of wine in the end… Just so I could rest a bit and charge up again*. *That was my trick*.

For some caregivers, the equilibrium between taking care and seeking distraction was found in couple activities. They organized activities (e.g., a small bicycle trip or having afternoon tea or coffee together) or they went on a short trip.

C1, C8 and C9 told about the journeys they took after hearing the diagnosis and how much they had enjoyed those times.

C1: *We still have done a lot and enjoyed many things together*. *We started planning big journeys when he began to get sick*. *I’m telling you*, *at the end of August*, *we travelled abroad*, *and in September*, *we still went biking in a neighboring country for the weekend*. *We combined those things and made the best out of it*.

However, not all journeys with a terminally ill patient turned out to be successful. Some could be considered as rather resilience-threating. C2, C3 and C5 talked about how much of a challenge it was to travel with their ill partners.

C3: *I’m telling you*, *a month before he passed away*, *we travelled abroad*, *against medical advice*. *But that was difficult*. [very emotional]. *I was so afraid*. *We stayed at the farm of some friends*, *something we had been doing for the past 15 years*. *Of the ten days we were there*, *he was doing well for three days*, *the rest of the time he had to stay in bed*, *so we couldn’t do anything*. *The days went by slowly*. *And uhm*, *when you’re in a strange country*, *and you don’t know the language*. *I was really scared at that time*. *It was like*: *What if something happens here*? *And that drainage didn’t go as it was supposed to go*. *He had a lot*, *and I mean a lot*, *of inconveniences and pain*.

#### Positivity and optimism

When caregiving was mainly characterized by positivity and optimism, the caregiver succeeded in giving positive meaning to the crisis. They called the caregiving experience a chance to give something back, to give the best of oneself or to work towards a better relationship.

C4: *It might be strange to say*, *but it does give the chance to show the best of yourself*.

A positive state of mind and the capacity of meaning-making through adversity was immediately linked to finding benefits in the caregiving process. Almost all participants discussed how their relationship with either their partner or family strengthened. Moreover, most of them ended up accomplished as they felt respected, important and trusted.

C3: *The fact is that your children appreciate you even more*. *They still tell me regularly*: *“But mom*, *what you did for our daddy*, *there are not many people capable to do such things*.*” That way*, *you get something back*, *right*?

C8: *Actually*, *how something so horrible* [as his partner who was dying from cancer] *can bring up such beautiful things*. *Yes*, *that’s just it*. *It was horrible and it still is*, *but… In the end*, *it was something beautiful*, *especially the moment she told me she had been happy*. *That gave me such a satisfied feeling*.

However, for some caregivers, finding meaning in the crisis was not obvious. They could not understand how cancer could attack someone who did not deserve it. C1, C2, C6 and C7 emphasized how healthy their partners had been before the cancer. Feelings of anger and helplessness clearly impeded their resilience.

C6: *And sometimes that brought up my anger of why it happened to us*. [husband’s name] *had*, *in my eyes*, *always lived healthy*, *he didn’t smoke and didn’t drink very often*. *Just a bit at a party or something but never too much*. *That makes you think*, *what did he do to deserve all this*? *He also didn’t weigh too much*. *I do suppose we lived quite healthily*.

Positivity and optimism were even expressed by attempts to fulfill the patient’s wishes. For example, the caregiving period of C2 and C8 was complete with social gatherings and activities: friends were invited, and the caregiver tried to create as many moments to cherish as possible. C3’s husband asked her to organize a goodbye party for more than a hundred guests. While C9 took care of his partner’s children during her hospital stays, because he knew how important this was for her.

C2: *And he*, *too*, *had such beautiful thoughts*. *For example*, *he said*: *“I want to connect people*.*” And it was true as well*, *the thought of connecting people*. *That’s just beautiful*, *isn’t it*? *So*, *well*, *that’s what he was doing*, *and I thought I had to follow him in his thoughts*, *right*? *I want to invite people to my home*, *even people who don’t know each other*, *but those who you think will like each other*. *And if they do*, *that’s so nice to see*. *He enjoyed all that as well*.

C9: *I know that for X*. *the children rank from one to three*, *and then from four to 99*, *and I am 100*. *I knew that was very important for her*, *the children’s lives should not be disturbed*. *So*, *what happens next*? *The clothes need to be washed*, *food must be ready on the table*, *and they needed the chance to go to school and practice a sport*. *So*, *I did everything I could to make this possible*.

Optimism was often expressed as sustaining a sense of hope and trust. Although the caregivers wanted to believe everything would turn out for the best, hope was often tempered by their realism: they hoped for a miracle but took into account that the chance of happening was extremely low.

C4: *You know that the results are coming*, *and you just need to wait constantly*. *And it has never*, *almost never*, *been good news*. *Sometimes a spectacular improvement*, *and then*, *you know*, *there is still hope*. *It has happened a few times that it indeed was very spectacular*. *Even the oncologist called us on our way back from the hospital*: *I’ve got the results*, *and they are better than expected*. *Then*, *it is worse again the next time*.

C1: *Enjoying*, *waiting*, *and we will stretch time hoping you can stretch it until*, *well*, *until medical science improves*. *And if he could have extended his life for a couple more years*, *then the immunotherapy probably would have been available for* [patients like] *him as well*, *uhm…*

For some caregivers, being able to maintain normality made them hopeful their life together could last for a very long time. C4 talked about the efforts he and his family did to guarantee the patient’s sense of connectedness with the family.

C4: *My wife was here at home*, *she has …* [cries]. *Her bed has been here six years*. *Meanwhile*, *she lived here in the middle* [around the kitchen]. *And the kids also cooked very often to get her involved in the smells and the noise*. *(…) The kitchen was*, *it was her kitchen*, *so yeah*. *In her free time*, *she made jam and stuff like that*. *But the kids took that over*. *That has been very important though*. *And I’m not a good cook*, *but I did cook a lot with the help of books*, *etc*. *Yeah*, *that was something important here*.

### Contextual resources

Themes in this category refer to the contribution of the caregivers’ context to resilience. The broader context encompasses two collateral systems continuously interacting throughout the caregiving process: firstly, a temporary, cancer-related context (e.g. oncologists, a palliative support homecare team and homecare nurses), and secondly, a system of relatives such as the family doctor (GP), children, other family, friends, and the patient.

Most caregivers did not hesitate to draw upon their context. However, others did not allow any involvement unless they explicitly asked for it or approved it. The patient’s diagnosis often made the context members take action by themselves without being asked, therefore significantly influencing the situation. Sometimes, they even used resources that were not employed before.

The way the context adapted to and influenced the situation was largely complex and individual. However, three distinctive, frequently occurring, and striking, dynamic context-related dimensions could be recognized: availability, meaningful relationships and the role of the patient.

#### Availability

Although the caregivers mentioned that they had never called the GP or anyone else in the middle of the night, they considered it of utmost importance to know that people were available at any time.

Regular visits by HCPs, were highly appreciated and were considered extremely supportive.

C6: *And when she* [GP] *came*, *she took her time*. *Once in a while she drank a cup of coffee with us*. *We never had the feeling we were a number and that we*, *you know*, *doctors live a busy life*, *but she always took time for him and*, *well*, *yeah*, *I do appreciate that*. *And I knew*, *she said*: *“if something’s wrong*, *day or night*, *you can always call me*.*” I felt supported*. *I never had to call her at night*, *but the fact she always said that*, *then you know it’s okay to do that*.

C6: *Uhm*, *so they have come to visit very often*. *They also said*: *‘‘If something’s wrong*, *just tell us*. *If we can help with something*, *we will*.*” You know you can go somewhere when you need a shoulder to cry on or when you want to tell your story*, *I could always go to someone*.

Some caregivers were afraid of being alone with their dying partner while others did not want the patient to be left alone, and they emphasized the importance of always having someone around they could trust.

C2: *The last 14 days*, *I told the children*: *There is one thing you need to do*: *I’d like to always have someone with me*. *I don’t want to be alone*, *just in case something happens*, *so I’m not alone because I was afraid of that*. *And uhm*, *they did*. *There was always someone*.

#### Meaningful relationships

Most participants talked about how their relationships altered after the diagnosis by becoming stronger, which are paramount in sustaining.

C7: *All this changed the relationships*, *right*? *That friend who did the night watch*, *has been my*, *well*, *badminton partner for the last ten years*. *But that relation has risen to another level*, *almost mother and daughter*.

Other relationships became more tenuous and were perceived as resilience threatening. This became very clear for C3, C4 and C5, when family members did not visit or postponed their visit until it was too late.

C3: *Well*, *I actually thought the biggest challenge was*, *uhm*, *making the family aware that they should try to spend as much time as possible with their brother*, *or son*. *Like*, *when you called asking if they could come visit*, *they always postponed*. *Time becomes a whole new dimension when*, *you know*, *like*, *it’s going to end*. *His siblings*, *for example*, *didn’t see him any the last year*. *That was difficult for me*.

Most caregivers were grateful for being surrounded by people who watched over them. C2, C3, C4 and C6 could share their emotions, difficulties and obstacles with their children and family. C2 called this sense of belongingness “the strength of the family”, an expression that was recognized by the other participants.

C2: *Luckily*, *we have a very strong bond with the children*, *three beautiful kids*. *They are children with a certain capacity but also who can show emotions*. *Luckily*, *we have talked a lot*.

C4: *Yes*. *The youngest has* …, *he’s very quiet*, *he’s a very quiet boy*. *But he has taken care of her*. *Just sitting right next to her and… yeah*. *You could feel the fact they all came home*. *And not a little*,*but six months*, *six months living back here*, *all five of us*. *Her sister has visited very often*, *her youngest sister who didn’t come regularly before because she lived far away*, *also visited very often*, *yeah*, *… We did it as a team*.

C5, C7 and C8 felt rather connected with their friends. In their stories, they emphasized how they could rely on them for advice or practical and emotional support. C7 compared her situation with a bible-parable.

C7: *The gospel was a parable of the paralyzed*, *of carrying and being carried*. *I carried my husband*. *But you can only carry once you’ve got enough carriers*. *Our entire entourage*, *acquaintances*, *friends and there were a lot of them*, *believe me*, *has*, *in the end*, *because of that disease*, *I could count the ones who remained on two hands*. *But the ones I still have*, *the ones I can count on two hands*, *they carry you through*.

#### The patient’s role

From the participants’ stories, the role of the patient and the quality of the relationship were found to be decisive elements in the caregiver’s resilience. Most caregivers spontaneously mentioned the quality of their relationship with the partner, talking about, for example, happy marriages, soulmates or by good times spent together. Others emphasized how their relationship was altered by the cancer, mostly getting better than it ever was before and considered this a benefit from caregiving. More than once, love was referred to as the most powerful prerequisite of resilience.

C2: *But yeah*, *be careful*, *I think you can only do such things when there is a lot of love*. *Then you can really do it*. *Really*. *And there was a lot of love*. *Yeah*, *then you really do it*.

C8: *Yeah*, *it’s like*, *we were together for almost 20 years*. *We do so much together*. *We really were two soulmates*. *So*, *yea*, *… Despite it being such a black period*, *it did bring us so much closer to each other*. *And that’s the most beautiful thing about all this*.

Knowing the patient was happy despite the cancer, supported the caregiver and resulted in feelings of accomplishment.

C2: *And then he wrote a poem in one night*. *We also put that on his prayer card*. *He never wrote*, *he never wrote poems*, *so*, *it really means a lot*. *(…)” I am and I will stay a very happy person”*. *And that means a lot to me*.

When the patient was optimistic and positive-minded, providing assistance seemed much easier for the caregiver, which in turn, left him/her optimistic too.

C4: *She was always very positive*. *Of course*, *that does a lot*, *right*? *It’s a story of interaction*. *She never complained*. *If you look at it that way*, *it’s a very easy way of caretaking when you’ve got a partner who also accepts it* [what you do for her] *and who handles that in a good way*.

C9: *I went along with her positivity*. *Because when your partner is so positive*, *you’re not going to tell her that it may only last six months or … No*, *then you just pull away the belief*, *you pull away all the hope from under her feet*. *You simply don’t do that*.

On the contrary, when the patient was in a depressed mood or was behaving inappropriately (e.g., drinking too much or not wanting to get out of his/her bed), the caregiver experienced higher stress levels and described this stage of the caregiving process as much more difficult.

C2: *We went to the beach for three weeks*, *and there he had a lot of those moments that he*, *yeah*, *that he struggled to get out of bed*. *That was very difficult*. *Yeah*, *that was not my husband at that moment*. *That really was not my husband*. *It did improve a little bit afterwards*. *But during that holiday*, *it was very difficult*.

## Discussion

Resilience was challenged by the partner’s diagnosis of incurable cancer. All participants made use of a set of interacting, caregiver-specific and context-related resources, facilitating a resilient process and leading to positive feelings and even personal growth. The caregivers demonstrated individual competences: adaptive flexibility, positivism, a sense of self-initiative, and adaptive dependency. They also relied on their context: cancer-related professionals, their family doctor, family members and friends. Context and situation were continuously interacting. The resulting dynamics were based on the context-availability, meaningful relationships and the patient’s role.

The concept of resilience has recently been reviewed in the elderly confronted with adverse events [60, 61], in adult patients with cancer and cancer-survivors [62], in caregivers of COPD patients [63], in mental health [64], in dementia caregiving [65], and following potential trauma [34]. From these reviews, resilience is generally conceptualized as the normative process of adapting well, or as growth in the face of adversity or after a PTE [64]. Frameworks resulting from the reviews emphasize the importance of individual resilience traits, talents, or skills, and the resulting coping strategies [65], and the role of the context [34, 60, 63, 64]. They are underpinned by our findings confirming that resilience trajectories result from the interplay between individual caregiver resources, comparable with the described resilience traits, and context resources. However, the underlying themes are related to the caregivers’ life experiences [62, 65]. Personal resources identified in the reviews confirm the original themes included in the resilience scale of Wagnild et al. (1993): equanimity, self-reliance, perseverance, meaningfulness and existential aloneness [62, 66, 67]. However, depending on the studied phenomenon, situation-specific and more dynamic personal resources are added. For example, in elderly research: generativity, hardship and experiencing giving [60]; in patients with cancer: sense of confidence, mastery, self-transcendence, self-esteem, capacity for negotiating, managing and adapting [62]; in dementia caregiving: personal mastery, self-efficacy and positive coping [65]; and motivation, hope, humor and self-determination in mental health research [64]. Our study adds the following aspects: sense of self-initiative, adaptive flexibility, adaptive dependency and positivism as specific caregiver resources for middle-aged partners of patients with incurable cancer.

All the reviews mentioned above accentuate the importance of contextual factors in facilitating resilience. The availability of social support, ability to access care and availability of economic resources are all recognized as resilience supporters in the elderly [60]. Social support, meaningful relationships and community resources are mentioned as resilience-enhancing in patients with cancer and cancer-survivors [62]. Developing social support networks, maintaining balanced relations and collaboration with HCPs support resilience in caregivers for patients with COPD. Research in mental health has shown that drawing on existing support networks, becoming a contributing member of one’s community, connecting affectively with friends and family, and having meaningful relationships enabling a sense of belonging, are the most important context resources [64]. Our study endorses the results of the reviews and expands them by emphasizing the importance of the availability of both cancer-related professionals and long-term relatives, meaningful relationships and, of course, the role of the patient.

Resilience in adult cancer caregiving has scarcely been studied [48, 49]. Hwang et al. (2018) approached the subject from a quantitative point of view. Resilience was found to be associated with good health status, increased patient performance and social support [49]. Since our study had a qualitative approach, comparisons between the studies should be considered with caution. Our study endorses the importance of social support and of the patient’s performance. However, according to our participants’ stories, the influence of the context on resilience reaches beyond social support, encompassing the meaningfulness of relationships. Moreover, the dynamics resulting from interactions between context, caregiver and situation are complex and not consistently resilience-supporting. For example, visits by family and friends are mostly perceived as emotionally supporting and their advice is welcomed. However, for some caregivers, those visits are considered as time-consuming and too confrontational with the reality of impending death. Additionally, their well-intentioned advice makes them feel insecure.

The accordance between the advanced cancer patient’s mood and the caregiver’s performance has been studied before, albeit principally in the context of distress and burden [68, 69] and seldom in the context of mental health [70]. When patients with advanced cancer meet the criteria for mental disorders (e.g., anxiety or distress), their caregivers are eight times more likely to develop psychological distress and vice versa [68, 69]; and the declining mental well-being of the patient is associated with the worsening mental health of the caregiver [70]. From our findings, we have arguments to confirm mutuality and reciprocity in the patient’s and caregiver’s mood, albeit from the opposite point of view: a positivily minded patient boosts caregiver resilience. Nevertheless, HCPs should avoid the trap of putting the responsibility for the caregiver’s resilience on the patient. In fact, the patient being in a negative mood was experienced as a threat to resilience and should be properly addressed by HCPs. Above all, a positive reciprocal interaction between the patients and their partners was found to be resilience-supporting.

In a qualitative inquiry, Roen et al. (2018) explored how HCPs, as part of the caregiver’s context, could promote resilience [48]. Their results globally reflect our findings about the importance of the temporary, cancer-related context being available, taking enough time to listen, giving advice, and informing what to expect when the patient dies. However, they did not examine the parallel, long-term context system existing among family, friends and the GP. We found this to be of utmost importance in supporting resilience, particularly by the meaningfulness of the relationships.

The findings of our study reflect and extend those of a previous study conducted by Totman et al. (2015). They have listed the threats of taking care of a patient with cancer at home, hereby focusing on the psychological complexity of emotional challenges, and organized them into four existential conditions: responsibility, isolation, death and the need to find meaning [50]. From our participants’ stories, similar challenges appeared. However, we emphasized how our participants tried to overcome those threats and the resources they could reply on to cope. Responsibility and isolation were mostly addressed through involvement and availability of the context, while difficulties of talking about death and dying were either avoided or were addressed by giving positive meaning. Furthermore, our participants found meaning both in the caregiving situation and in their relationships. These findings are in line with the results of the systematic review conducted by Pottie et al. (2014), stating that meaningfulness of care and social support are highly associated with the caregiver’s enhanced psychological well-being while taking care of a palliative relative [21].

Researchers agree that interconnected and consistent elements foster resilience in general: biological, individual and environmental factors [62, 71]. In this study, the focus was on caregiver-specific and context-related resources and then applied to the understudied domain of middle-aged partners of patients with incurable cancer. Although specific resources could contribute to resilience, this study underpins the assumption of resilience being much more complex than a simple balance of threats and resources [62]. Competences that are identified as facilitators of the resilient process sometimes seem contradictory. For example, a sense of self-initiative, where the caregiver strives to control the situation and is aware of his/her existential aloneness is considered as much a facilitator as adaptive dependency, meaning that the caregiver searches for and accepts all support offered. Furthermore, resources that seem to be resilience facilitators for one caregiver sometimes hamper resilience in someone else. Visits from friends and family, for example, are mostly experienced as emotionally supportive and resilience facilitating. However, for some caregivers those visits seem to be too much of a confrontation with pending death or are regarded as meddlesome. Coping resulting from caregiver and context resources can sometimes appear maladaptive (e.g., withdrawing when feeling sad or refusing visits from friends and family), but nonetheless are resilience facilitating. Moreover, all our participants took advantage of a variety of different competences, depending on emerging events. This confirms the assumption that resilience is dependent on the situation and may alter if circumstances change [72].

Specialists often associate resilience with other salutogenic concepts (sense of coherence, positive health and post traumatic growth (PTG)). Antonovsky (1993) explains successful coping with stressors by reaching a sense of coherence, positing that the challenge is seen as comprehensible, manageable and meaningful [73]. From our data, the importance of a sense of coherence is reflected in the caregivers’ resilience resources. Our caregivers’ sense of self-initiative helps them to understand and control the situation, while positivity leads to finding meaning in caregiving. Managing the situation is mainly achieved by flexibly adapting one’s lifestyle and by adaptive dependency characterized by seeking and accepting help from others.

A second salutogenic concept, positive health, is defined as: *the ability to adapt and to self-manage*, *in the face of social*, *physical and emotional challenge* [74]. Resilience is *a process facilitating appropriate adaptation to a challenging situation and leading to a healthy functioning that goes beyond the absence of disease* [75]. Consequently, the interplay between caregiver and context resources described in our study can facilitate the achievement of positive health. PTG refers to enhanced personal strength, appreciation for life, relations with others, new possibilities and spiritual-existential change [76]. Hence, PTG can be thought of as the ideal outcome of resilience. In this study, some caregivers’ stories indicate that PTG was achieved. However, this was mostly the case in participants who had the least caregiver resources to rely on and whose resilience was facilitated primarily by their context resources, suggesting that PTG is positively associated with context resources but correlates negatively with caregiver resources. Our findings could be explained by the theorem of Eicher et al. (2015) and Levine et al. (2009). They suggest that personal growth from resilience might be distinct from PTG. Resilience is supposed to mitigate the impact of trauma and to protect against psychological wounds [62, 77].

### Strength, limitations and suggestions for further research

A qualitative approach with an inductive thematic analysis provides valuable information and insights into phenomena that have not yet been extensively studied [55, 56]. Our analysis indeed revealed some situation-specific resources (e.g., adaptive dependency, sense of self-initiative and meaningfulness of relationships) that were not yet listed as resilience resources under other circumstances.

This study has several limitations, however, since a double selection bias cannot be excluded completely. Firstly, the participants were recruited from caregivers who were supported by palliative homecare teams, and therefore, probably had a better chance of having more positive outcomes than caregivers who were not supported by a specialized team. Secondly, the group of participants could be biased towards caregivers who reported a positive outcome from their experience. The response rate was relatively low, likely suggesting that only caregivers who adapted well returned the invitation to participate. It is not unlikely that our findings were influenced by this with a predominance of resilience resources over threats, and as such, could evoke the impression that caregiving inherently leads to a resilient trajectory. It should always be kept in mind that some caregivers lack the resources for a resilient trajectory and are at risk for physical or psychological disorders.

Moreover, it is remarkable that all of our participants had a higher education. Although no evidence was found confirming a relationship between education and resilience, cautiousness is needed when transferring our results to lower educated caregivers.

Because no standards exist for measuring resilience as a process, and consequently, resilience can only be inferred from a positive outcome, a retrospective approach seemed most appropriate [62]. This approach implies risks of bias: firstly, we should be aware of the risk of recall bias [78]. The caregivers’ accounts are based on their memories, and thus, can be adapted over time. However, this risk of bias was addressed by explicitly and repeatedly asking for difficulties, challenges and facilitators during caregiving. Secondly, there is the risk of ‘overgeneral memory’, meaning that the memory is biased by the mood state by recalling negative events faster than positive experiences. This phenomenon has been studied extensively in affective disorders and after having witnessed trauma. Although it is suggested that this bias does not occur when trauma is not complicated by a PTSD or by prolonged emotional disturbance, it cannot be excluded that positive mood or resilience leads to idealized memory [79]. Thirdly, the death of the patient could be considered a second PTE with an unknown influence on the resilience trajectory. Although a study by Morin et al. (2017) establishes that repeated PTEs do not influence resilience [80], others suggest that repeated stressors (e.g., a change in prognosis or bad news) do affect the process outcome either by strengthening resilience through a so-called steeling effect or by rendering more vulnerable through sensitization [81, 82]. As a result, the influence of repeated PTEs, even as the influence of experienced benefits on the resilience trajectory is unclear, and longitudinal studies are needed to unravel this ambiguity.

This thematic analysis reveals different resources for a resilient trajectory that are specific to the cancer caregiving situation. However, it does not provide insight into the underlying processes. Questions remain unanswered as how and why some caregivers manage to establish resilience supporting capacities after a fatal diagnosis, while others give up and lose hope. How is it that some caregivers succeed in establishing a supporting context with friends, family and professionals on whom they can rely while others either reject all offered help or experience it as an extra burden? Our study provides insight into the interacting resources but not in the interactions themselves. Realist research, a method which seeks to establish what works, for whom, in what circumstances, in what respects and to what extent and why, may provide the answers [83].

Similarly, are the complex interactions of the caregiver’s context adapting to and influencing the situation and vice versa. Although three common themes discussing prerequisites of the context in enhancing resilience could be discovered from our data, many questions about the interdependencies and relations of the system elements remain unanswered. A “Complex Adaptive System (CAS)” refers to a context-system consisting of humans continuously interacting with one another in a non-linear, complex way. Approaching the caregiver’s context as a CAS could assist in a better understanding of the complex interconnectivity, the role of the patient as vital to the caregiver’s context, and the overall systems-level behavior rather than individual conduct [83–85].

Outcome trajectories following a PTE and their predictive factors have been studied extensively in the post-loss period, uncovering distinct trajectories, with resilience being the most common outcome [24, 28, 31, 32, 43, 86]. However, to our knowledge, the pre-loss period, starting from the diagnosis of advanced until the death of the patient, has not yet been explored in that way.

When this loss is due to cancer, it will mostly be preceded by at least one PTE, namely the patient being diagnosed with cancer in an advanced, incurable stage. Our participants discussed about how they created moments to cherish or how they treated the patient and gave the best of themselves in order to not have to feel guilty afterwards. It would be of interest to know if a resilient trajectory during caregiving protects against depression during the bereavement period or prepares the caregiver for the post-loss period.

Our study revealed caregiver specific facilitators and threats for the resilient process we inferred from a positive outcome. Nonetheless, we cannot claim any predictive value from these facilitators and threats nor can we assess the nature of the trajectories. Therefore, our results should be complemented by a longitudinal survey focusing on the identification of different trajectories and the predictive value of the resources we discovered.

### Impact

Earlier research revealed that hospice-based caregiver interventions that targets the enhancement of positive aspects of caregiving and that promote problem-solving and meaning-based coping strategies, are much more promising than those that which target the reduction of burden [21]. Our study can inspire researchers through the development of resilience-targeting interventions for advanced cancer caregivers.

When assisting caregivers, the emphasis should be on guiding them to reach a status of positive health by adapting flexibly to the new situation. This would avoid medicalization of resilience threats as having difficulties to find meaning or temporary anxiety. Taking care of the caregiver in a positive health-oriented way with attention to both caregiver and context resources and the complex interactions between them, should be embedded in daily clinical practice. Therefore, this study can benefit psychologists and clinical health care professionals in optimizing undergraduate and postgraduate training programs.

### Conclusion

When supporting partners of patients with advanced cancer, it is desirable to assist them towards a resilient trajectory throughout the caregiving period. A resilient trajectory results from a complex interplay between situation-dependent intrinsic and extrinsic resources. To build resilience in middle-aged partners of patients with advanced cancer, HCPs should pay attention to the caregivers’ resources (sense of self initiative, positivity, adaptive flexibility and adaptive dependency). They should be aware of being part of the caregiver’s context, a complex adaptive system that can be either resilience-supporting or -threatening. Therefore, the HCP may be attentive not only to the existence or absence of intrinsic and extrinsic resources but even to every slight change in the interaction between the resources, since every resilience facilitator can suddenly turn into a threat. Moreover, it is recommended not to underestimate the unique role of the patient. Indeed, there seems to be a very strong reciprocity of feelings and emotions between the patients and their partners. A positive minded patient makes it easier for the partner to deal with the adverse situation. However, when the patient’s mood drops, the HCP should be attentive because this can often be considered a resilience threatening challenge for the partner.
